# Comparison of the effectiveness of anchoring needles and coils in localizing multiple nodules in the lung

**DOI:** 10.1186/s12890-022-02192-8

**Published:** 2022-11-01

**Authors:** Ya-Yong Huang, Tao Wang, Yu-Fei Fu, Yi-Bing Shi, Wei Cao, Ju-Pan Hou

**Affiliations:** Department of Radiology, Xuzhou Central Hospital, 199 South Jiefang Road, Xuzhou, China

**Keywords:** Localization needle, Coil, Pulmonary nodule, Wedge resection

## Abstract

**Background:**

Recently, a new type of pulmonary nodule positioning needle has been adopted clinically. We aimed to evaluate the efficacy and safety of a new type of localization needles compared with coils for the simultaneous localization of multiple pulmonary nodules guided by computed tomography (CT) prior to video-assisted thoracoscopic surgery (VATS).

**Materials and methods:**

From January 2021 to March 2022, 87 pulmonary nodules from 40 patients were localized using the new localization needle. From January 2020 to December 2020, 68 pulmonary nodules in 31 patients were localized using coils. The relative outcomes were compared.

**Results:**

The success rate of pulmonary nodule localization in the needle group was 97.7% while that in the coil group was 98.5%. In the needle group, the time needed to locate the first nodule was significantly shorter than in the coil group (10.9 min vs. 17.2 min, P = 0.001). Moreover, the time needed per patient was also significantly shorter for the needle group compared with the coil group (23.7 min vs. 30 min, P = 0.017). The incidence of pneumothorax in the needle group was 25.0% vs. 12.9% in the coil group (P = 0.204). The rate of pulmonary hemorrhage in the needle group was 40.0% vs. 32.3% in the coil group (P = 0.502). The success rate of VATS wedge resection was 100% in both groups.

**Conclusion:**

Both disposable pulmonary nodule localization needles and coils are safe and effective for CT-guided localization of multiple pulmonary nodules of the same stage prior to VATS. However, the use of needles is time-saving compared with the use of coils. The coil localization may exhibit better safety than needle localization.

## Background

Due to an increase in people’s awareness of health examination options and the improvement of medical imaging methods, the detection rate of pulmonary nodules has increased significantly, and many patients are diagnosed with multiple pulmonary nodules [1, 2]. An excisional biopsy is an option for determining the nature of pulmonary nodules, especially in anxious patients [3]. Video-assisted thoracoscopic surgery (VATS) has been widely used in clinic. Compared with thoracotomy, it brings less invasion to patients [4]. It allows the maximization of the preservation of normal lung tissue during pulmonary nodule resection [2]. However, it is difficult to accurately observe and palpate the target nodule during surgery, which limits the application of VATS in the case of small pulmonary nodules [5]. To overcome this problem, a variety of preoperative pulmonary nodule localization methods are used in clinical practice [6]. Localizers usually involve coils, hook wires, methylene blue, and radiolabeling agents. [7]. Recently, a new type of pulmonary nodule positioning needle, which is an improved device for the hook thread positioning needle, has been clinically adopted [8]. There have been previous reports on the use of coils to locate multiple pulmonary nodules [1, 4], but there are no reports on the use of novel localization needles targeting multiple pulmonary nodules.

This retrospective study aimed to evaluate the efficacy and safety of a novel localization needle compared with the use of a coil for simultaneous localization of multiple pulmonary nodules under computed tomography (CT) guidance prior to VATS.

## Methods

### General data

This retrospective study was approved by the Institutional Ethics Review Board and informed consent was waived.

**Fig. 1:**
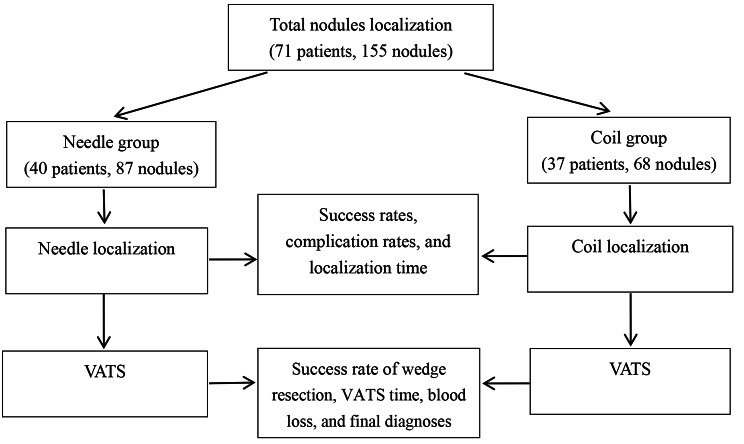
The flowchart of this study

From January 2021 to March 2022, 40 patients with a total of 87 pulmonary nodules received CT-guided positioning of the new localization needle before VATS in our center. From January 2020 to December 2020, 31 patients with a total of 68 pulmonary nodules underwent CT-guided coil localization before VATS in our center (Fig. 1). In a collaborative effort, radiologists, thoracic surgeons, and oncologists identified and localized the patient’s pulmonary nodules that were to be resected.

The inclusion criteria were: (1) The patients were investigated once and the number of VATS pulmonary nodules was ≥ 2; (2) The maximum diameter of the located pulmonary nodule was ≤ 30 mm; (3) The distance between the nodule and the adjacent pleura was ≤ 30 mm; (4) Patients did not have surgical contraindications; (5) Patients with unilateral pulmonary nodules because single-stage surgery for bilateral pulmonary nodules may carry the risk of serious complications [2].

The exclusion criteria were: (1) Complete calcification nodules; (2) Pulmonary nodules with reduced volume after follow-up, because this kind of nodules were usually given continuing follow-up; (3) Patients with underlying diseases such as severe emphysema and pulmonary fibrosis; (4) Patients with coagulation disorders.

### Equipment and materials

Computed tomography was done using an Optima CT 680 scanner (GE Healthcare). The tube voltage was 120 kV, the tube current was 100–150 mA, and the layer thickness was 2.5 mm. The locator in the needle group was a 100 mm, long 20G diameter pulmonary nodule positioning needle (Shengjie Kang, Ningbo, China). Each needle consisted of 5 parts: puncture needle, tri-color suture, 4 hook anchor, push rod, release buckle, and a protective tube. The locator in the coil group was a 70 mm long, 0.038-inch diameter tower-type microcoil loaded in the cannula (Cook, Bloomington, IN, USA) puncture needle (PRECISA, Roma, Italia) 18G/100mm.

### Localization procedure

#### Needle group

**Fig. 2:**
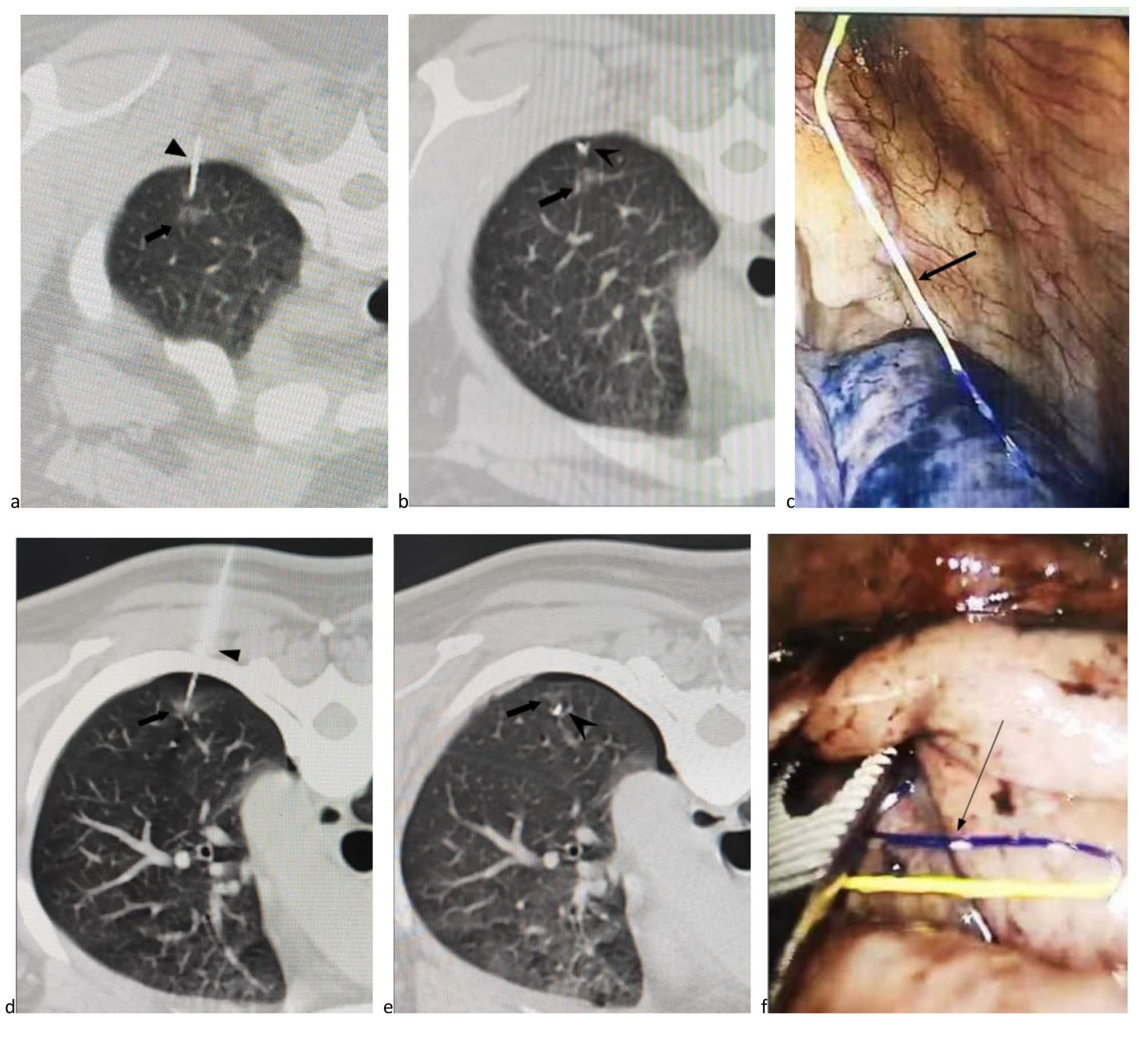
A patient displaying 2 lung nodules at the left upper and lower lobes. (a) A lung nodule (arrow) was located at left upper lobe and the puncture needle (arrowhead) was inserted near the nodule. (b) The anchor of the needle (arrowhead) was placed near the lung nodule (arrow). (c) The localization suture (arrow) was visualized during the VATS. (d) A lung nodule (arrow) was located at the left lower lobe and the puncture needle (arrowhead) was inserted near the nodule. (e) The anchor of the needle (arrowhead) was placed near the lung nodule (arrow). (f) The localization suture (arrow) was visualized during the VATS.

The positioning procedure was completed under CT guidance. The patient’s position and needle insertion path were designed according to the preoperative images. A CT scan was performed on the patient, and the puncture point and puncture path were determined according to the scanned image. The procedure included disinfection, use of a sterile towel, subcutaneous application of 2% lidocaine, and pleural local anesthesia. A disposable pulmonary nodule positioning needle was used to gradually puncture the predetermined area (the needle tip was inserted within 10 mm of the lesion), followed by the removal of the release buckle. Next, the push rod was pressed to release the 4-hook anchor, the puncture needle was pulled out, and the rod was pushed so that the three-color line remained on the puncture path. A second CT scan was performed to observe the positional relationship between the hook anchor and the pulmonary nodule and to determine whether there were any complications (Fig. 2). The procedure was then repeated for other target lung nodules.

### Coil group

**Fig. 3:**
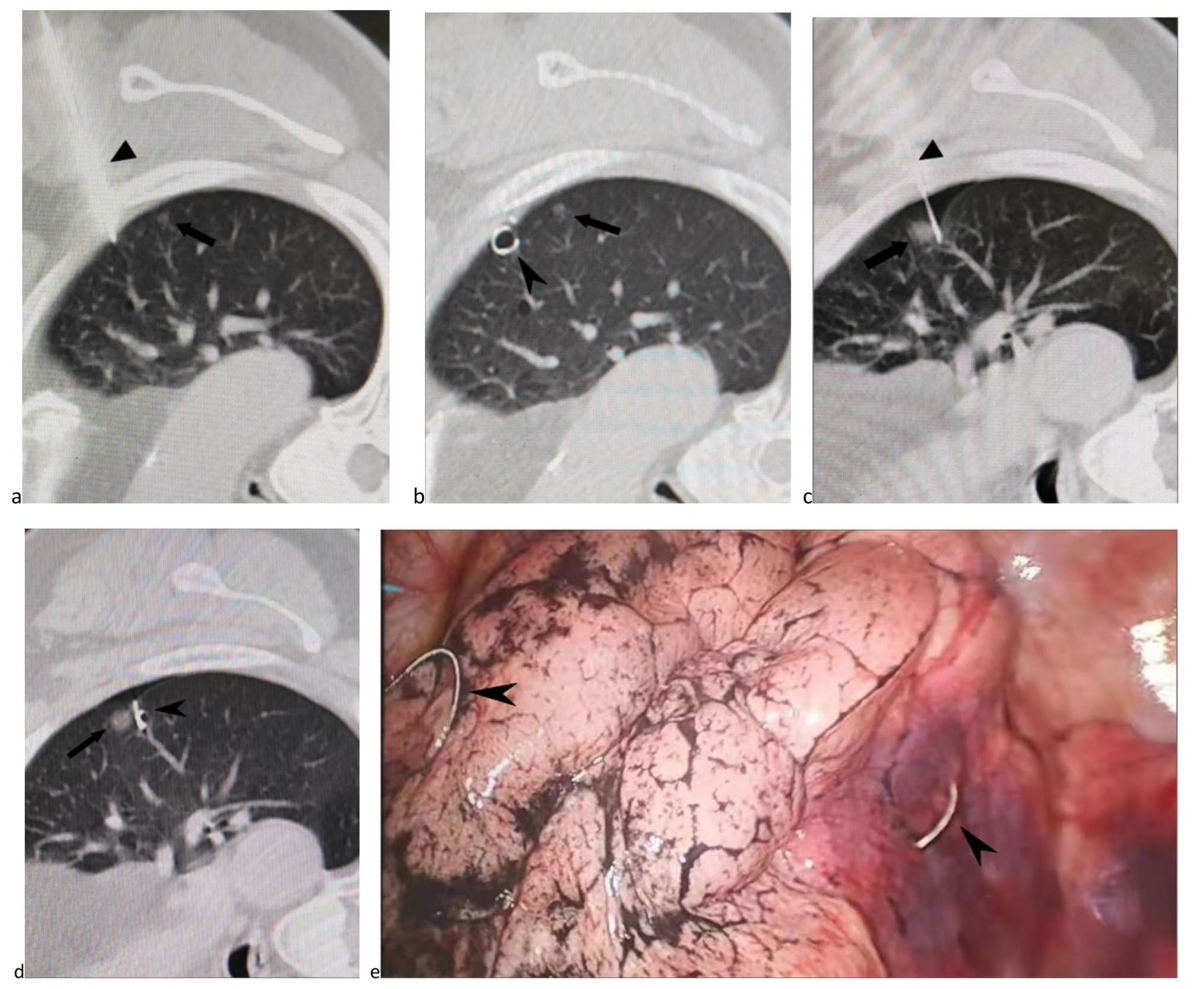
A patient displaying 2 lung nodules at the left upper lobe. (a) A lung nodule (arrow) located at left upper lobe and the puncture needle (arrowhead) was inserted near the nodule. (b) The coil (arrowhead) was placed near the lung nodule (arrow). (c) Another lung nodule (arrow) was located at left upper lobe and the puncture needle (arrowhead) was inserted near the nodule. (d) The coil (arrowhead) was placed near the lung nodule (arrow). (e) The 2 coils (arrows) were simultaneously visualized during the VATS.

The step-by-step procedure of percutaneously puncturing the positioning needle to the predetermined area for the coil group was similar to that of the needle group. The CT scan was repeated to determine the position of the needle tip, the coil with the needle core was pushed and released next to the pulmonary nodule, while the tail of the coil was left outside of the visceral pleura. A CT re-examination confirmed the positions of the coil, the pulmonary nodule, the tail, and the pleura. If the positions were not as expected, a second coil was inserted as a supplement. If the expected requirements were met, the puncture needle was pulled out to end the positioning procedure, and the patient was assessed for possible complications (Fig. 3). The above procedure was then repeated for other target lung nodules.

### Thoracoscopic resection

All VATS were performed within 24 h of completion of CT-guided localization. Wedge resection was performed by observing the tail of the coil remaining outside the visceral pleura and the caudal line connected to the hook anchor as a marker. A pathologist performed a rapid pathological evaluation of the resected pulmonary nodules. A pathological diagnosis of benign tumor, adenocarcinoma in situ (AIS), or mini-invasive adenocarcinoma (MIA) concluded the surgery. A pathological diagnosis of invasive adenocarcinoma (IAC) was followed by segment/lobar resection and lymph node dissection.

### Observations and definitions

Localization success was defined as the ability to observe either the tail of the coil remaining outside the visceral pleura, or the tail of the tricolor attached to the hook anchor during the VATS procedure. A successful wedge resection was defined as the identification of a target pulmonary nodule within the resected lung parenchyma [1]. Localization time was defined as the time from patients lying on the bed of CT to completeness of insertion of the localization materials [2]. The VATS time was defined as the time from first incision to wound closure [2]. Severe pulmonary hemorrhage was defined as needle tract or perilesional hemorrhage > 2 cm in width [9]. Massive pneumothorax was defined as a pocket of air with a width of > 2 cm in the pleural space on axial CT images [9].

Observation indicators included gender, age, number of nodules, location, density, maximum diameter, distance from adjacent pleura of pulmonary nodules, localization success rate, time required for nodule localization, procedure-related complications, VATS duration, and final pathological results of pulmonary nodules.

### Statistical analysis

Continuous variables were expressed as means, and categorical variables were expressed as percentages (%). Continuous variables were compared using an independent sample *t*-test, and categorical variables were compared using χ^2^ test and Fisher’s exact test. P < 0.05 was considered statistically significant. All analyses were performed using SPSS 16.0 (SPSS Inc, Chicago, IL).

## Results

### Baseline data

The coil group included 68 pulmonary nodules from 31 patients, and the needle group included 87 pulmonary nodules from 40 patients (Table 1). There were no significant differences in the gender and age of the patients between both positioning groups. Furthermore, there were no significant differences in nodule number, location, density, maximum diameter, or distance from the adjacent pleura between the two groups.

**Table 1:** Comparison of patients’ and pulmonary nodules’ baseline data between 2 groups

	Needle group	Coil group	P value
Patients number	40	31	-
Age (years)	55.3 ± 8.9	55.8 ± 9.8	0.814
Gender(Male/Female)	11/29 (27.5%/72.5%)	15/16 (48.4%/51.6%)	0.07
Emphysema	6(15.0%)	5 (16.1%)	1.000
FEV _1.0_ /FVC (%)	79.4 ± 5.8	78.0 ± 7.3	0.388
Pulmonary nodules number	87	68	0.896
Patients with 2 nodules	34 (85.0%)	26 (83.9%)	-
Patients with ≥ 3 nodules	6 (15.0%)	5 (16.1%)	-
Nodules diameter (mm)	7.4 ± 2.7	6.9 ± 4.0	0.333
Nodule-pleura distant (≤/> 10 mm)	57/30 (65.5%/34.5%)	50/18 (73.5%/26.5%)	0.284
Natures of the nodules			0.281
Solid	18 (20.7%)	17 (25.0%)	
Mixed GGN	6 (6.9%)	9 (13.2%)	
Pure GGN	63 (72.4%)	42 (61.8%)	
Sites of the multiple pulmonary nodules			0.767
Right upper lobe	35 (40.2%)	22 (32.4%)	
Right middle lobe	6 (6.9%)	6 (8.8%)	
Right lower lobe	23 (26.4%)	18 (26.5%)	
Left upper lobe	15 (17.2%)	12 (17.6%)	
Left lower lobe	8 (9.3%)	10 (14.7%)	

FEV _1.0_ /FVC: forced expiratory volume at 1 s/forced vital capacity; GGN: ground-glass nodule

### Positioning-related data

Positioning-related data are shown in Table 2. In the needle group, the localization success rate was 97.7% (85/87). In two patients, localization of the second pulmonary nodule with the needle tip resulted in the folding of the visceral pleura into the lung. Therefore, the needle tip could not effectively penetrate the lung parenchyma, resulting in localization failure. The success rate of localization in the coil group was 98.5% (67/68). In one patient of the coil group, following the localization of one pulmonary nodule, the tail end of the coil was not indwelled outside the visceral pleura, resulting in localization failure. Overall, there was no statistically significant difference in the positioning success rate between the two groups. No localizer displacement occurred in any successfully localized nodules in either group. The time needed for the positioning of the first nodule was significantly shorter in the needle group compared with the coil group (10.9 min vs. 17.2 min, P = 0.001) and likewise the per patient positioning time was significantly shorter in the needle group compared with the coil group (23.7 min vs. 30 min, P = 0.017).

**Table 2:** Localization-related results

	Needle group	Coil group	P value
Technical success based on nodules	97.7% (85/87)	98.5% (67/68)	1.000
Technical success based on patients	95.0% (38/40)	96.8% (30/31)	1.000
Duration of localization procedures (min)			0.001
Duration of first nodule localization (min)	10.9 ± 5.0	17.2 ± 6.8	0.001
Duration of localization of one patient (min)	23.7 ± 7.1	30.0 ± 9.2	0.017
Position change	19 (47.5%)	14 (46.7%)	0.845
Complications			
Pneumothorax	25.0% (10/40)	12.9% (4/31)	0.204
Lung hemorrhage	40.0% (16/40)	32.3% (10/31)	0.502
Severe hemorrhage	5.0% (2/40)	0	0.501

In the needle group, the rate of pneumothorax was 25.0% (10/40), and the rate of pulmonary hemorrhage was 40.0% (16/40). One patient developed massive pneumothorax after localization of the first nodule, requiring pleural aspiration before the second nodule localization was completed. The rate of pneumothorax in the coil group was 12.9% (4/31), and the rate of pulmonary hemorrhage was 32.3% (10/31). There was no statistically significant difference between the two groups in the incidence of pneumothorax and pulmonary hemorrhage, and no severe pulmonary hemorrhage occurred at any location.

### Data on thoracoscopic resection

The success rate of VATS wedge resection of pulmonary nodules in both groups was 100%, and no patients required conversion to thoracotomy (Table 3). For the 1 nodule that failed to localize in the coil group, a wedge resection of the lung was successfully performed by palpation of the coil. In the needle group, one failed nodule was a solid pulmonary nodule with a diameter of about 15 mm, and a wedge resection of the lung was successfully performed by palpation combined with the position of the previously localized pulmonary nodule in this patient; in the needle group, another nodule that failed to locate was a ground-glass nodule with a diameter of about 10 mm. During the VATS procedure, it was successfully located by observing the bleeding point left by the puncture needle in the visceral pleura in combination with the position of the previously located pulmonary nodule in the patient, followed by lung wedge resection.

**Table 3:** Surgical types and final diagnoses

	Needle group	Coil group	P value
Technical success of wedge resection	100%	100%	-
Duration of VATS (min)	106.1 ± 65.8	125.3 ± 43.5	0.165
Types of resection			0.226
Wedge resection	77 (88.5%)	64 (94.1%)	
Wedge resection + segment/lobectomy	10 (11.5%)	4 (5.9%)	
Intraoperative blood loss(ml)	48.2 ± 48.1	58.7 ± 43.9	0.349
Final diagnosis			0.001
IAC	11 (12.6%)	5 (7.4%)	
MIA	30 (34.5%)	6 (8.8%)	
AIS	15 (17.2%)	24 (35.3%)	
Atypical hyperplasia	4 (4.6%)	14 (20.6%)	
Benign	27 (31.1%)	19 (27.9%)	

VATS: video-assisted thoracoscopic surgery; MIA: mini-invasive adenocarcinoma; AIS: adenocarcinoma in situ; IAC:Invasive adenocarcinoma

The number of nodules diagnosed as IAC by rapid intraoperative pathology in the needle group and coil group was 11 (12.6%) and 5 (7.4%), respectively. Of these, 10 (11.5%) patients from the needle group and 4 (5.9%) patients from the coil group underwent additional segment/lobectomy and lymph node dissection. Segment/lobar resection was not performed in 1 patient from each group because those patients were unable to obtain sufficient reserves to support continued lung function.

There was no significant difference in resection type (P = 0.226), average VATS duration (P = 0.165), and intraoperative blood loss (P = 0.349) between the two groups. However, there was a statistically significant difference in the final pathological results of pulmonary nodules between the two groups (P = 0.001). The MIA ratio was the highest in the needle group, while the AIS ratio was the highest in the coil group, each followed by benign tumors in both groups. The proportion of multiple pulmonary nodules in the patient with inconsistent final pathological results was 55.0% (22/40) in the needle group and 54.8% (17/31) in the coil group, a difference that was not statistically significant (Table 4, P = 0.989).

**Table 4:** The final pathological results of multiple pulmonary nodules in the same patient are inconsistent

The final pathological results of multiple pulmonary nodules in the same patient are inconsistent	Needle group	Coil group	P value
Yes	22 (55.0%)	17 (54.8%)	0.989
No	18 (45.0%)	14 (45.2%)	

## Discussion

CT-guided percutaneous pulmonary nodule localization can effectively help thoracic surgeons to improve the success rate of VATS and can assist pathologists in quickly and accurately locating target pulmonary nodules during surgery. While there are many kinds of devices used in localization, it has previously been reported that the use of coils yields optimal clinical results [6, 10]. Disposable pulmonary nodule localization needles present a new type of localization device [8]. At present, there are few reports on their clinical efficacy, especially in the case of multiple pulmonary nodules.

In the current study, the success rate of the coil group in localizing multiple pulmonary nodules was 98.5%, which was similar to that reported by Teng et al. [11]. The success rate of the needle group in this study was 97.7% (85/87) and the difference with the coil group was not statistically significant. There were two pulmonary nodules in the needle group that we failed to locate, both of which were the second nodules to be located. In those two cases, following the location of the first pulmonary nodule, a small amount of pneumothorax appeared, and the visceral pleura was folded on itself when the needle tip was re-punctured, preventing the puncture needle from effectively entering the lung parenchyma. This situation has been reported in many studies [2, 4, 12]. After carefully analyzing the reasons, we propose that: (1) The lung tension decreased following the occurrence of pneumothorax [4, 13]; (2) The needle tip of the positioning needle is a hollow wedge-shaped structure; (3) There were other tissues such as thick blood vessels near the target pulmonary nodule. In the coil group, we also failed to locate one nodule, and the coil tail left outside the visceral pleura could not be observed during VATS, although the procedure was successfully completed by finger palpation.

**Table 5:** Predictors of pneumothorax

	Univariate analysis			Multivariate analysis		
	Hazard ratio	95% CI	P value	Hazard ratio	95% CI	P value
Age	0.973	0.916–1.033	0.362			
Gender						
Male	1					
Female	1.359	0.414–4.462	0.613			
Minimum diameter of the multiple nodules	1.328	0.978–1.804	0.069	1.323	0.953–1.836	0.095
Maximum nodule-pleura distance of the multiple nodules	1.064	0.992–1.141	0.081	1.053	0.973–1.140	0.200
Number of the nodules	1.028	0.304–3.469	0.965			
Emphysema	1.356	0.314–5.850	0.683			
FEV _1.0_ /FVC (%)	0.995	0.913–1.084	0.913			
Position change	0.602	0.196–1.850	0.376			
Localization material						
Needle	1			1		
Coil	0.346	0.099–1.205	0.096	0.606	0.146–2.513	0.490

**Table 6:** Predictors of lung hemorrhage

	Univariate analysis			Multivariate analysis		
	Hazard ratio	95% CI	P value	Hazard ratio	95% CI	P value
Age	1.048	0.991–1.109	0.103			
Gender						
Male	1					
Female	0.997	0.375–2.648	0.994			
Minimum diameter of the multiple nodules	1.130	0.859–1.485	0.383			
Maximum nodule-pleura distance of the multiple nodules	1.048	0.985–1.115	0.135			
Number of the nodules	0.869	0.303–2.496	0.794			
Emphysema	1.167	0.320–4.251	0.815			
FEV _1.0_ /FVC (%)	1.002	0.932–1.078	0.957			
Position change	1.575	0.607–4.085	0.350			
Localization material						
Needle	1					
Coil	0.476	0.180–1.263	0.136			

The incidence of pneumothorax in the needle group and coil group was 25.0% (10/40) and 12.9% (4/31), respectively, which was similar to that reported elsewhere [2, 13]. Some studies have pointed out that a longer positioning time and a puncture path through the interlobar fissure are independent predictors of pneumothorax [2]. In our study, although there was no statistically significant difference in the incidence of pneumothorax between the two groups, the incidence of pneumothorax in the needle group was about twice that in the coil group, which may be clinically significant. This study is a retrospective analysis of previous data, and the sample size of patients is limited. We will carry out relevant prospective studies in the future work to further analyze and demonstrate.

The occurrence of pneumothorax may affect the localization of other nodules in the ipsilateral lung [13, 14]. In this study, following the occurrence of pneumothorax, puncturing was more difficult in the needle group than in the coil group. However, the positioning time of the first nodule in the needle group and the positioning time per patient were both significantly less than those in the coil group, because the anchor release procedure of the positioning needle was easier than the coil placement.

The incidence of pulmonary hemorrhage was similar in the two groups, and no massive hemorrhage occurred. The diameter of the single-use pulmonary nodule positioning needle was 20G. The thinner the puncture needle, the lower the risk of vascular injury in the lung. However, its locator has 4 anchors, which creates some tension when it is released, and there is a risk of injury to the adjacent blood vessels. Thus, ideally, the operator should release the anchor hook in an area without blood vessels. Coils consist of an interventional material applied to blood vessels. The material is soft, and after release it is circular, and the outer circumference is also wrapped with villi, which creates a certain hemostatic effect [4]. Therefore, the incidence of pulmonary hemorrhage in both groups was very low.

The success rate of VATS wedge resection was 100% in both groups. There were no significant differences in the mean duration of VATS, mean intraoperative blood loss, and surgical resection between the two groups, which were all similar to previous reports [2, 4]. Both localizers were shown to be effective for VATS wedge resection.

There were differences in the final pathological results between the two groups. The occurrence of MIA was highest in the needle group, and the AIS ratio was highest in the coil group. Both were in the early development stage of IAC, and the clinical treatment methods are the same, so the final results of this study are not different. We also observed, that in some cases the pathological results of multiple pulmonary nodules from the same patient were different, and the proportion of such cases in the needle group and the coil group was as high as 55.0% and 54.8%, respectively, which reflected that the patients with multiple pulmonary nodules were evaluated for a single nodule. It also indicates the importance of single-stage localization and wedge resection of multiple pulmonary nodules [12].

This study has certain limitations. First, this was a retrospective study and second, this was a single-center study. Therefore, a larger, multicenter prospective study on the effectiveness of single-use pulmonary nodule localization needles for the CT-guided localization of multiple same-stage pulmonary nodules prior to VATS should be conducted in the future.

## Conclusion

Our results show that: (1) Single-use pulmonary nodule localization needles and coils are both safe and effective for the CT-guided localization of multiple same-stage pulmonary nodules prior to VATS; (2) The use of single-use pulmonary nodule localization needles is time-saving compared with the use of coils. (3) The coil localization may exhibit better safety than needle localization. However, this point should be validated further.

## Data Availability

The data that support the findings of this study are available from the corresponding author upon reasonable request.

## List of abbreviations

AIS: adenocarcinoma in situ
CT: computed tomography
IAC: invasive adenocarcinoma
MIA: mini-invasive adenocarcinoma
VATS: video-assisted thoracoscopic surgery
